# A two-stage surface defect segmentation method for wind turbine blades based on Deeplabv3+

**DOI:** 10.1038/s41598-025-33608-0

**Published:** 2025-12-29

**Authors:** Xin Li, Jinghe Tian, Xinfu Pang, Li Shen, Haibo Li, Zedong Zheng

**Affiliations:** 1 Key Laboratory of Energy Saving and Controlling in Power System of Liaoning Province, Shenyang Institute of Engineering, Shenyang, 110136 P R China; 2 School of Computing and Mathematical Sciences, University of Leicester, Leicester, LE1 7RH UK

**Keywords:** Defect segmentation, deeplabv3+, DenseASPP, Wind turbine blade, Wind energy, Computational science, Electrical and electronic engineering

## Abstract

A two-stage segmentation model based on improved Deeplabv3 + is proposed for segmenting Blade regions and defects in complex environments. Accurate segmentation is critical for timely maintenance and safe operation of wind turbines. The model consists of Blade-Deeplabv3 + and Defect-Deeplabv3+, collectively named BD-Deeplabv3+. In the first stage, Blade-Deeplabv3 + segments the Blade from the background using the Atrous Spatial Pyramid Pooling module to extract multi-scale features and suppress background interference. The resulting segmented Blade is then input to the second stage. In this stage, Defect-Deeplabv3 + extracts multi-scale features and refines boundaries of surface crack, hole, and spalling defects. DenseASPP replaces the original ASPP, employing densely connected dilated convolutions to enhance multi-scale feature fusion and improve semantic representation and boundary accuracy for minor defects. Experimental results show that the mean intersection over union for Blade segmentation reaches 98.97%, and for defect segmentation reaches 94.25%. Finally, Blade defect severity is quantified using the ratio of defect area to Blade area, enabling more reliable maintenance planning.

## Introduction

### Literature review

With the surge in global demand for clean energy, the installed capacity of wind power continues to rise, with more turbines being installed worldwide. As a representative of clean energy, wind energy has abundant reserves and significant cost-effectiveness and shows unique advantages in efficient development and sustainable utilization^1^. As the core component of energy capture, wind turbine blades are exposed to extreme environments such as wind-sand erosion, lightning strikes, hail, and corrosive acid rain for a long time, resulting in frequent surface damage^2,3^. Wind turbine blades are a pivotal component in wind turbines’ overall functionality, and they exhibit a high propensity for failure. The expenditure on material procurement, transportation logistics, design, and development accounts for about 15% -20% of the overall cost^4,5^. If surface defects such as scratches and holes are not detected in time, they may rapidly expand into structural cracks, threatening the safe operation of the unit^6,7^. The length of the blades is usually more than 50 m^8^. Manual inspection has significant potential safety hazards, and low efficiency and a high rate of missed detections are common issues with traditional inspection methods. Applying UAV technology to inspection operations significantly improves operational flexibility, reduces human resources investment, and ensures operational safety^9,10^. Aerial image detection based on drones faces the dual challenges of complex background interference, such as clouds and vegetation, and the scale diversity of defects from millimeter-scale holes to meter-scale damage.

In^11^, an improved UCD-YOLO model is proposed. The lightweight LCSP_SPPF module is designed to combine the CAM module to improve the efficiency of multi-scale feature fusion. At the same time, the Wise-IoU loss function is introduced to optimize the regression accuracy. Aiming at the problem that large wind turbine blades are challenging to detect comprehensively due to the limitation of the field of view, a method combining image stitching and an improved Unet network is proposed in^12^ to realize the fine detection of overall and local defects. In^13^, a two-stage segmentation model was proposed, and the blade region was successfully isolated by comparing the six models. The damage segmentation selects and optimizes the ResNet50-SegNet model, and the recall rate of damage segmentation is 82%. In^14^, the objective is to resolve issues concerning the early detection of damage to wind turbine blades. ResNet18 was used to replace the ResNet50 of the original DINO, and the ECA attention mechanism was added between the residual blocks to enhance the feature attention of the damaged area. In^15^, for the infrared image segmentation task of blades, a deep separable convolution block is introduced based on U-Net to improve the model’s ability to process fuzzy boundaries. In^16^, the encoder of U-Net was replaced by ResNet to enhance the feature extraction ability. ECA-Net and PSA-Net attention mechanisms are introduced to improve feature extraction and fusion capabilities. In^17^, a deep learning model based on U-Net architecture was constructed for pixel-level segmentation of surface defects of blades, achieving an accuracy of 96% in micro-crack detection. In^18^, hyperspectral imaging was used for the first time to detect the defects of blades. The experiment was limited to small samples, and the practical application effect was not verified. In^19^, the external attention module and ResNet101 backbone were introduced into DeepLabv3+, and the class imbalance problem in pavement disease segmentation was optimized by adaptive sampling. In^20^, for the concrete crack segmentation task, the Deeplabv3 + trunk is replaced by MobileNetv2, and DSC replaces the standard convolution to reduce the model parameters and calculation. In^21^, ResNet50 is used as the backbone network in Deeplabv3+, and DenseASPP and attention mechanism are introduced to improve the detection ability of small targets. In^22^, an improved Deeplabv3 + model was proposed. The channel attention module was introduced into the encoder, combined with the multi-level context attention mechanism, for the colonoscopy polyp segmentation task. In^23^, a skin cancer lesion segmentation method combining ViT and optimized Deeplabv3 + is proposed to enhance the localization of small lesions and the robustness of the model. In^24^, MST-DeepLabv3 + is proposed for semantic segmentation of remote sensing images. MobileNetV2 is used to replace the backbone, SENet, and transfer learning is introduced to improve the performance of small target recognition and edge segmentation and reduce the number of model parameters. In^25^, combined with binocular vision and improved DeepLabV3+, the IDAM module and ECA attention mechanism are designed for fine measurement of concrete cracks. In^26^, BEFUnet is proposed for medical image segmentation. A dual-branch encoder is designed to separately capture edge and body information, and a Local Cross-Attention Feature (LCAF) module is used to efficiently fuse features, enabling accurate segmentation of complex structures with irregular boundaries. In^27^, a novel multilayer multimodal fusion model (MultiFusionNet) is proposed for chest X-ray image classification. The model integrates features from different layers of ResNet50V2 and InceptionV3 architectures through a dedicated Fusion of Different-Sized Feature Maps (FDSFM) module, achieving significant performance improvements in pneumonia and COVID-19 detection tasks. A synopsis of associated research is delineated in Table 1.

**Table 1:** Summary of related work.

References	Wind turbine blade detection	Blade defect detection
^11^	-	Improved yolov5
^12^	U-Net(VGG16)	U-Net(VGG16)
^13^	SegNet(ResNet50)	Improved SegNet(ResNet50)
^14^	-	WTB-DINO
^15^	Improved U-Net	-
^16^	U-Net ( ResNet50 ) with attention mechanism added	-
^17^	-	U-Net
^18^	-	hyperspectral imaging
This study	Deeplabv3+(Xception)	DenseAspp-Deeplabv3+(Xception)

### Research gap and contributions

Although several studies have investigated blade defect detection under complex outdoor backgrounds, current approaches still present notable limitations. Existing research primarily relies on object detection frameworks such as YOLO and SSD, which provide only bounding-box-level localization. These methods are unable to extract precise defect contours and therefore cannot quantify crack length, defect area, or morphological characteristics. Consequently, important diagnostic information required for evaluating defect severity is lost. Segmentation-based approaches offer pixel-level defect delineation; however, most recent models—including improved U-Net, PSPNet, and variants of Deeplabv3+—typically adopt a single-stage architecture. When applied to field-captured wind turbine Blade images, these models struggle to simultaneously suppress complex backgrounds and extract small, fine-grained defects. Background elements such as soil, sky, and vegetation dominate the early feature encoding, while defects such as crack, hole, and spalling exhibit large scale variations and unclear boundaries, making them difficult to detect within a unified model.

To address these limitations, this study proposes BD-Deeplabv3+, a decoupled two-stage segmentation framework tailored for wind turbine Blade inspection. Unlike single-stage or generic segmentation architectures, the proposed method separates the tasks of background suppression and fine-grained defect extraction into two dedicated models. The first stage, Blade-Deeplabv3+, focuses solely on Blade-region extraction and removes complex background interference. The resulting clean Blade mask is passed to the second stage, Defect-Deeplabv3+, which concentrates exclusively on defect segmentation. DenseASPP is incorporated to strengthen multi-scale representation and preserve subtle defect characteristics, forming a processing pipeline that existing single-stage networks do not provide.

The main contributions of this work are as follows:

(1) A two-stage segmentation framework, BD-Deeplabv3+, is developed for Blade surface defect detection under complex backgrounds. The first stage extracts the Blade region, while the second stage targets only the defect areas, enabling more accurate segmentation of both structures.

(2) In Blade-Deeplabv3+, the ASPP module of Deeplabv3+ (Xception) is used to extract multi-scale contextual features, effectively suppressing interference from soil, sky, and vegetation and ensuring reliable Blade segmentation.

(3) In Defect-Deeplabv3+, DenseASPP replaces the original ASPP module to enhance multi-scale aggregation and retain fine-grained details of crack, hole, and spalling defects. Dense dilated convolutions gradually expand the receptive field while maintaining continuous scale coverage.

The remaining components of this paper are arranged as follows: The second section introduces the specific steps of two-stage blade defect segmentation, including Blade-Deeplabv3 + blade region segmentation, Defect-Deeplabv3 + blade surface defect region segmentation, and blade surface defect severity grading evaluation. The third section continues the simulation experiment. The fourth section is a summary.

## Two-stage blade defect segmentation method

### Problem description

Wind turbine blade defect detection is the core link to ensure wind power generation equipment’s reliable and safe operation. Still, there are some problems in the existing methods under complex backgrounds: (1) Traditional detection algorithms such as threshold segmentation and edge detection, the distinction between wind turbine blades and background is easily disturbed by uneven illumination and texture diversity of background images, resulting in incomplete extraction of blade body. (2) Although the defect location method based on target detection (such as YOLO series) can quickly identify the defect location, it can only output the regional positioning anchored by the bounding box, and it is difficult to accurately quantify the area ratio of the defect area to the blade body. The ratio of the defect area to the blade body area is a key indicator for assessing the severity of the defect. Therefore, a two-stage image segmentation framework based on Deeplabv3 + is proposed. In the first stage, the encoder-decoder structure is combined with space pyramid pooling (ASPP) to segment the blade and background accurately. This approach mitigates the impact of intricate background elements on defect detection. In the second stage, fine-grained segmentation is performed in the blade area to distinguish the normal blade area from the defect area, and the defect severity classification evaluation is realized by calculating the ratio of the defect pixel area to the total blade area.

### Strategic structure

**Fig. 1:**
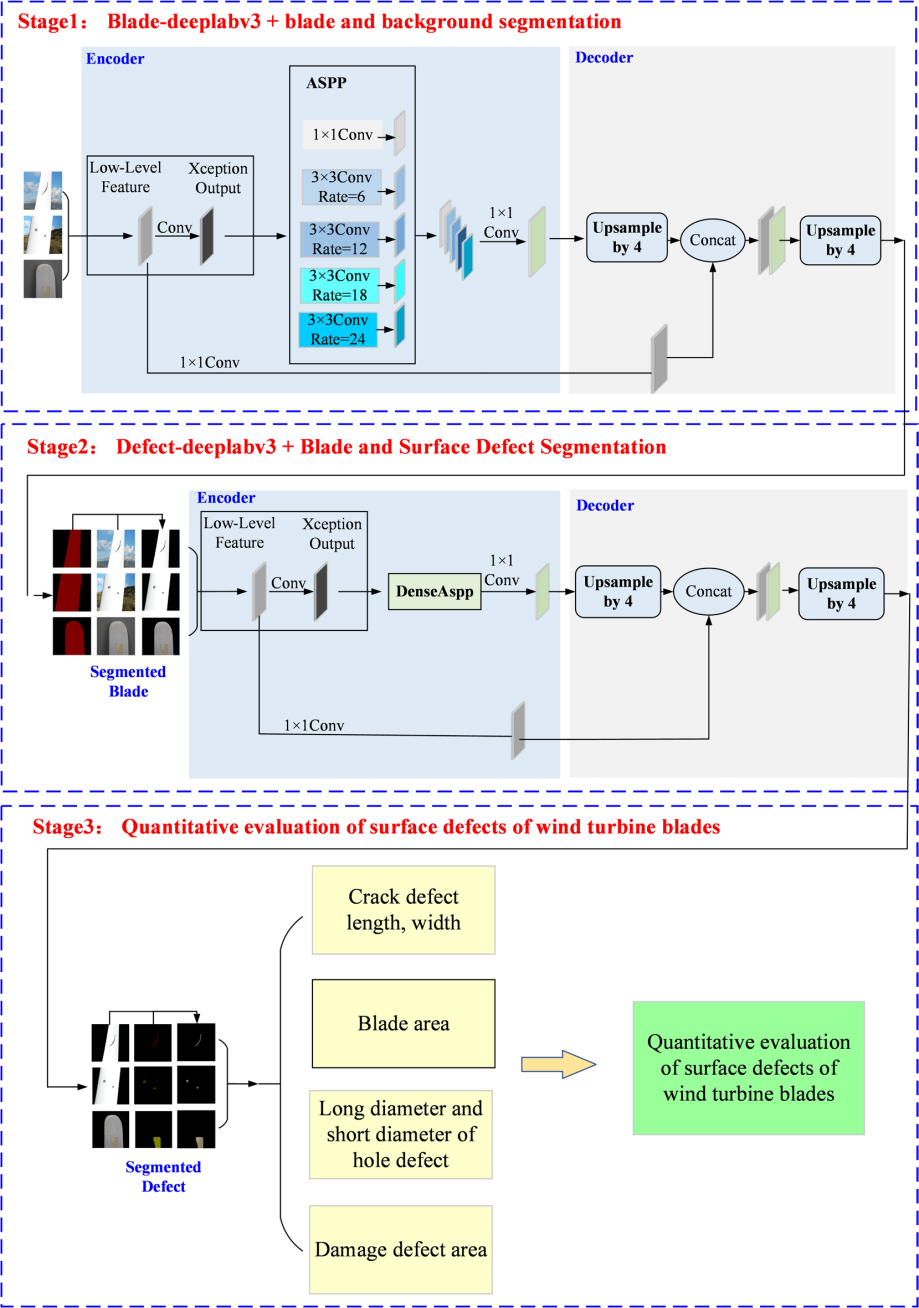
Two-stage blade defect segmentation method strategy structure.

The two-stage wind turbine blade defect segmentation method consists of Blade-Deeplabv3 + and Defect-Deeplabv3+, and the strategy structure is shown in Fig. 1. (1) Wind turbine blade region segmentation. The original blade image containing complex backgrounds such as the earth and the sky is input into Blade-Deeplabv3 + to cut out the blade area. (2) Segmentation of blade surface defect regions. The blade image without background segmented in the first stage is used as the input of Defect-Deeplabv3 + in the second stage, effectively avoiding background influence on the defect area and improving the segmentation accuracy.

### Blade-Deeplabv3 + Blade region segmentation method

Extracting the accurate edge of the blade area from the complex background is the premise of the next step. The Deeplabv3 + image segmentation algorithm with multi-scale feature fusion ability is used to locate the wind turbine blade area, and the blade area of the wind turbine is segmented from the background containing various interference factors. Avoid confusion between subsequent blade surface defect segmentation tasks and interference factors in the background.

Deeplabv3 + is based on DeepLabv3 and is an extended version of DeepLabv3. The encoder uses DeepLabv3’s Atrous Spatial Pyramid Pooling (ASPP) module to capture multi-scale context information through atrous separable convolution^28^. A simple and efficient decoder module is added to optimize the boundary segmentation of the target by fusing deep semantic features and shallow detail features. Xception is the backbone feature extraction network of Deeplabv3 + and uses deep separable convolution to convolve the space of each channel separately^29^. Xception is as shown in Algorithm 1.

**Algorithm 1:**
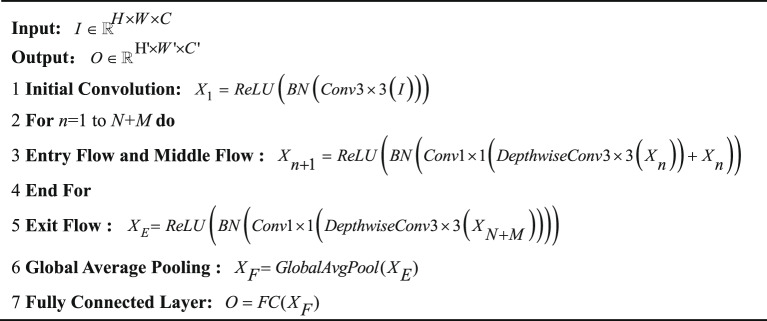
Xception Network.

To adapt to the image segmentation task, Deeplabv3 + modified part of the structure of Xception. To extract feature maps of any resolution using atrous separable convolution, all maximum pooling operations are replaced by deep separable convolution. Deep convolutions are each succeeded by Batch Normalization and ReLU activation. Batch normalization is shown in Eqs. (1–4).

1 $${\mu _A}=\frac{1}{n}\sum\nolimits_{{i=1}}^{n} {{x_i}}$$

2 $$\sigma _{A}^{2}=\frac{1}{n}{\sum\nolimits_{{i=1}}^{n} {\left( {{x_i} - {\mu _A}} \right)} ^2}$$

3 $${\tilde {x}_i}=\frac{{{x_i} - {\mu _A}}}{{\sqrt {\sigma _{A}^{2}+\varepsilon } }}$$

4 $${y_i}=\gamma {\hat {x}_i}+\beta$$

Where *n* is the batch size, ${\mu _{\mathrm{A}}}$ is the mean of the features, $\sigma _{{\mathrm{A}}}^{2}$ is the variance of the features, $\varepsilon$is a non-zero decimal, $\gamma ,\beta$ are learnable parameter.

**Fig. 2:**
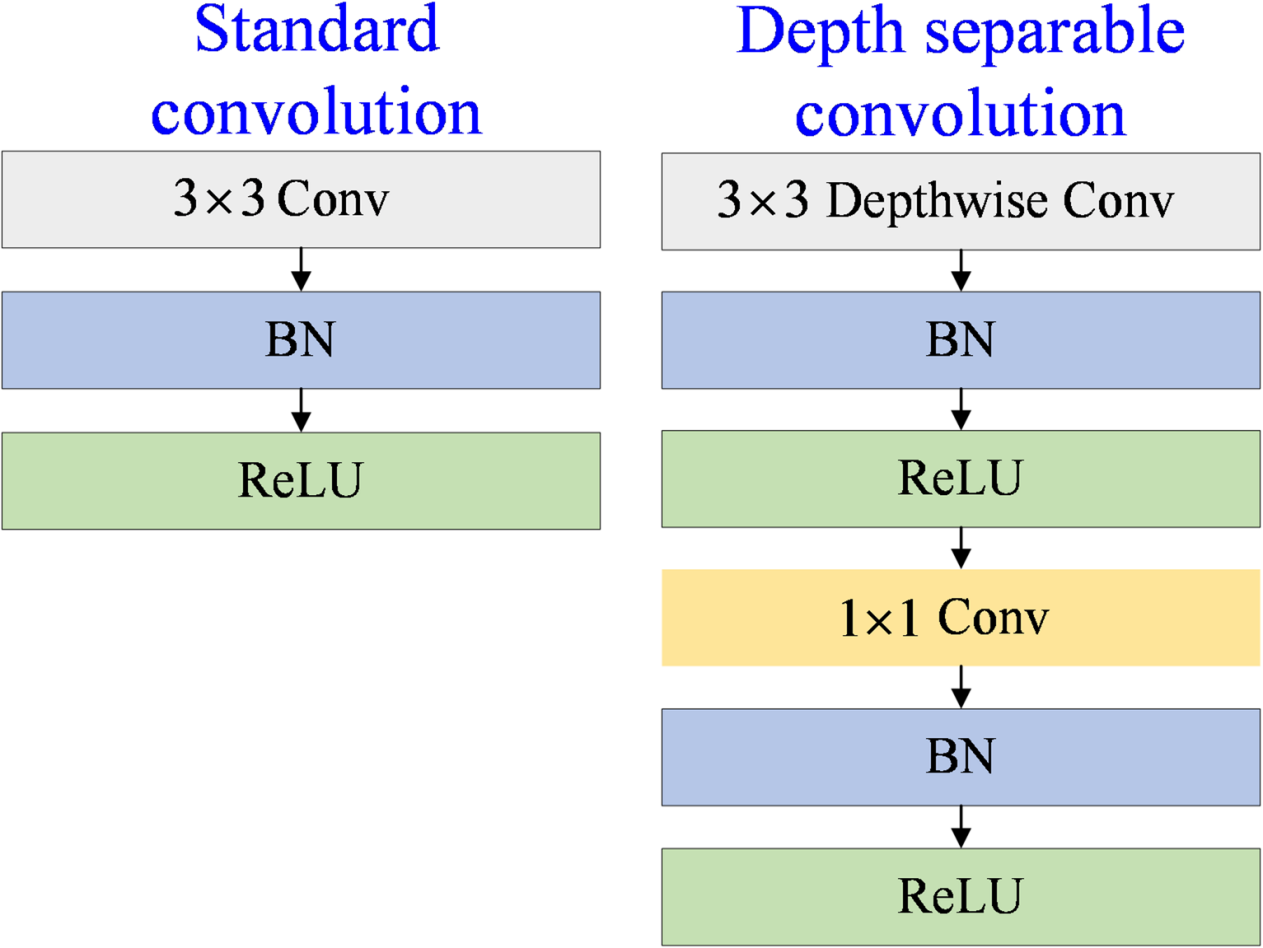
Standard convolution and depthwise separable convolution structure diagram.

Depthwise separable convolution splits standard convolution into a depthwise operation across channels and a pointwise operation. The deep convolution is processed independently by channel-by-channel. Each single-channel convolution kernel corresponds to a channel of the input feature map, so the number of output channels is strictly equal to the number of input channels. Point-by-point convolution uses a convolution kernel to process the input feature map. Since the width and height of the convolution kernel are both 1, this operation only weights the channel dimensions and does not change the spatial resolution of the feature map. The architecture of the depthwise separable convolution is depicted in Fig. 2.

The Atrous convolution is one of the core technologies of the Deeplabv3 + model. Introducing the expansion rate inserts spaces between the convolution kernel elements to expand the receptive field without increasing the parameters, thereby effectively capturing multi-scale context information. For the case of two-dimensional signals, on each position *i* of the output feature map *y*, the formula for applying the Atrous convolution to the input feature map *x* using the convolution filter $\omega$ is as shown in Eq. (5).

5 $$y[i]=\sum\limits_{{k=1}}^{K} {x[i+r * k]} \omega [k]$$

Where *r* is the expansion rate, *y* is the output, *x* is the input, $\omega (k)$ is the *k*th parameter of the filter, and *K* is the size of the filter.

In Deeplabv3+, the Atrous Spatial Pyramid Pooling (ASPP) module is the core component of the encoder part. Its primary function is to enhance the semantic understanding ability of the model to complex scenes through multi-scale feature extraction. ASPP contains multiple branches: one standard convolution, three Atrous separable convolutions, and one global pooling. Each Atrous separable convolution branch uses different expansion rates (*r* = 6, 12, 18). These branches perform convolution operations on the input feature map to extract features in different receptive field ranges. The larger expansion rate can cover a wider area and capture the global context; the smaller expansion rate focuses on local details. The global average pooling branch compresses the feature map into a vector and then recovers to the original space size by upsampling. This branch provides global context information to help the model understand the semantic layout of the entire scene. Traditional convolutional neural networks usually gradually reduce the resolution of feature maps by pooling or step convolution, which leads to the loss of spatial details. ASPP avoids this problem by Atrous separable convolution, which allows the receptive field to be expanded without increasing the number of parameters, so it can maintain the high resolution of the feature map while still covering the larger context area. This is crucial for image segmentation because accurate pixel-level classification is required. By adjusting the expansion rate of the Atrous separable convolution, the output step size of the feature map can be controlled so that more dense features can be extracted when computing resources allow.

### Defect-Deeplabv3 + blade defect region segmentation method

**Fig. 3:**
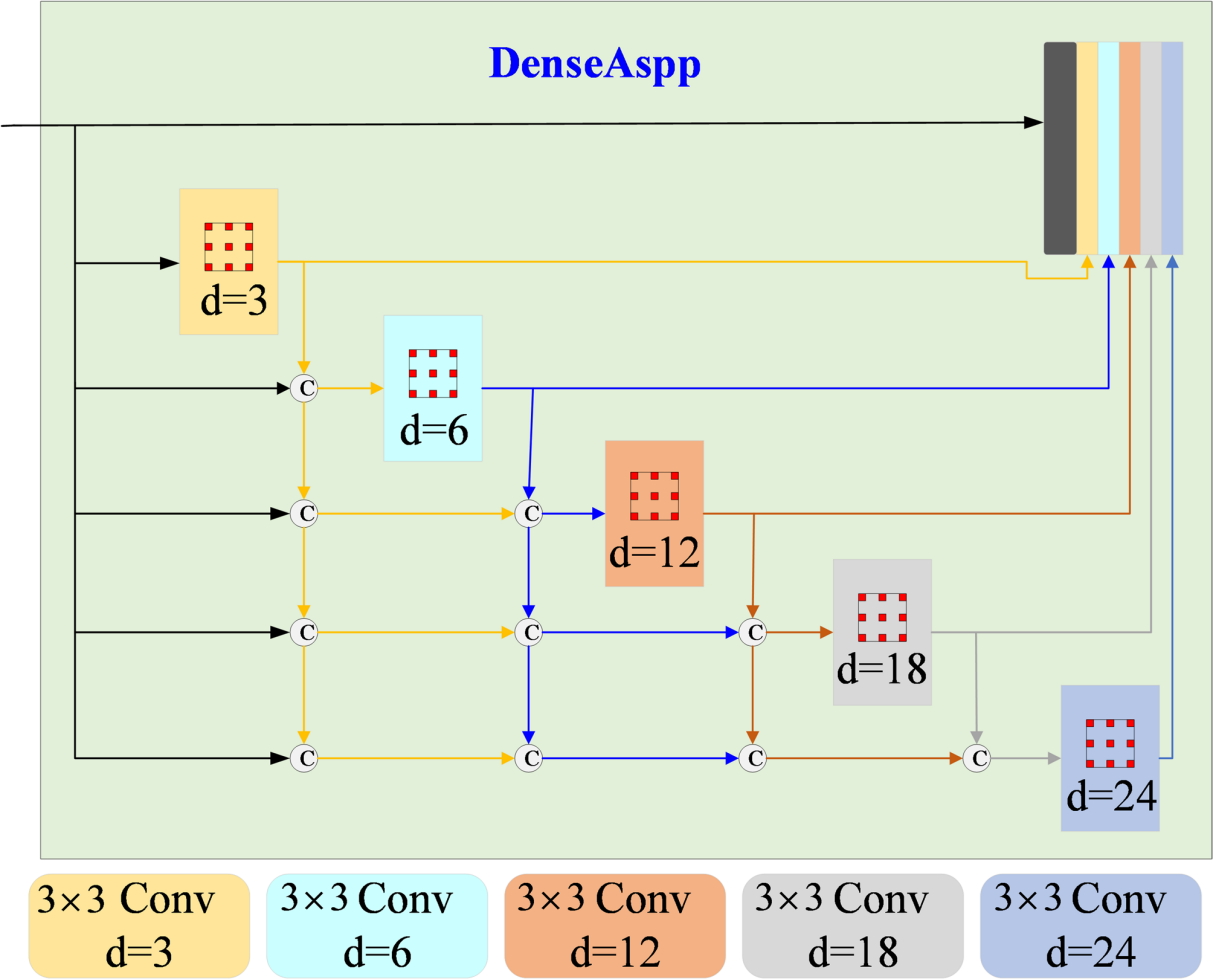
DenseAspp structure diagram.

​​​​ The surface defects of blades, such as slender cracks, small holes, and large-area damage, differ in morphology and scale. Most existing studies focus on detecting crack defects and do not identify multi-category defects^30^. The ASPP module used in the original Deeplabv3 + extracts multi-scale features through dilated convolution with a fixed expansion rate. Still, it is insufficiently adaptable to defect types with extreme size differences. After replacing the ASPP module with DenseASPP, through more densely connected multi-branch dilated convolution, more dense scale features can be dynamically fused, covering from pixel-level details to global semantic information to more accurately identify defects of different sizes. DenseASPP uses frequent cross-layer connections and reuses high-resolution features of low-level layers, which helps small target segmentation and avoids loss of details^31^. The segmentation of relatively large damage defects requires global context information, while minor cracks and holes require local details. By densely superimposing convolutions with different expansion rates, DenseASPP can simultaneously construct the relationship between local defects and the overall structure of the blade and reduce the missegmentation caused by interference factors. The DenseAspp structure diagram is shown in Fig. 3.

DenseASPP adopts a cascaded dilated convolution layer, and its expansion rate increases layer by layer. DenseAspp is as shown in Algorithm 2. The lower layer of the network is configured with a small expansion rate to perceive local details, and the top layer captures the global context through a significant expansion rate. In the process of layer-by-layer transmission, the output of each layer will be spliced with the original input features and the lower layer results, and the integrated features will be input into the upper layer network. The final output of the multi-scale feature map is generated by the fusion of convolution operations with different expansion rates. This design can construct a feature pyramid with dense coverage of multiple receptive fields through a small number of levels, significantly improving spatial information representation efficiency. Each hole convolution layer in DenseASPP can be represented by Eq. (6).

6 $${y_l}={H_{k,{d_l}}}([{y_{l - 1}},{y_{l - 2}}, \cdot \cdot \cdot ,{y_0}])$$

Where ${d_l}$ represents the expansion rate of the *l*th layer, $[ \cdot \cdot \cdot ]$ represents the cascade, and $[{y_{l - 1}},...{y_0}]$ represents the feature map formed by connecting the outputs of all previous layers.

**Algorithm 2:**
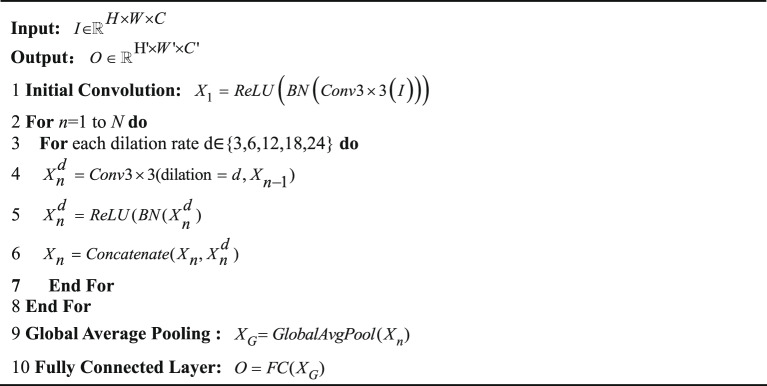
DenseAspp.

### Grading evaluation method of blade surface defect severity

Grading evaluation can accurately judge the severity of defects and avoid the subjective error of traditional qualitative judgment. Through numerical evaluation, maintenance or replacement plans are formulated in advance to prevent serious accidents such as blade fracture and collapse and ensure the safety of staff and the stable operation of equipment. Quantifying the data to distinguish the defect level can reduce unnecessary downtime maintenance time, reduce operation and maintenance costs, and maximize power generation revenue. The quantitative indexes of cracks are selected as length and width. The defect severity of the hole is evaluated by the diameter of the long axis and the short axis. The damage is measured according to the defect coverage area due to the large proportion of irregular area. To achieve consistency in the quantitative evaluation of defect severity, the area of various defect types is uniformly selected as the quantitative index. It is necessary to calculate the area of surface defect area and blade overall area and establish the quantitative benchmark of defect proportion. Finally, the blade risk level is evaluated according to the overall proportion of various defects. The defect area-to-blade area ratio is calculated as the basis for grading the severity of wind turbine blade defects. Its calculation formula is shown in Eq. (7).

7 $$P=\frac{{{S_{{\mathrm{Defect}}}}}}{{{S_{{\mathrm{Blade}}}}}}$$

Where ${S_{{\mathrm{Defect}}}}$ is the area of the defect area segmented in the second stage, ${S_{{\mathrm{Blade}}}}$ is the area of the blade segmented in the first stage, and *P* is the ratio of the defect to the blade.

## Experimental verification and analysis

### Experimental environment

The software and hardware equipment conditions required for the experiment in this paper are shown in Table 2.

**Table 2:** Experimental equipment conditions.

Computer software and hardware	Version / Model
GPU	Nvidia RTX 4090(24GB)
CPU	Intel Xeon Platinum 8352 V
operating system	Ubuntu 22.04
Python	3.8
Pytorch	1.10
CUDA	11.3

### Parameter setting

All models use a unified parameter configuration to eliminate the interference of hyperparameter differences. The learning rate $\eta$= 1e-4, *momentum* = 0.90, *epoch* = 150, the batch size *n* = 4, and the optimizer is selected as Adam. A summary of these parameter setting is provided in Table 3.

**Table 3:** Parameter setting.

Parameter	Value
Learning rate	1e-4
Momentum	0.90
Epoch	150
Batch size	4
Optimizer	Adam

The traditional cross-entropy loss may cause the model to pay too much attention to negative samples, thus ignoring positive samples, especially those that are difficult to detect. To solve the class imbalance in training and the unevenness of positive and negative samples, the Focal loss function and the Dice loss function are used. The Focal loss function is shown in Eq. (8). The overlap between predictions and actual labels is quantified using the Dice loss function. The model is optimized by minimizing the difference between the two, as shown in Eqs. (9) and (10).

8 $$Focal\_Loss({p_t})= - {\alpha _t}{(1 - {p_t})^\gamma }\log ({p_t})$$

Where ${p_t}$ is the correct category probability predicted by the model, ${\alpha _t}$ is the category weight, and $\gamma$ is the adjustment factor. Increasing $\gamma$ will make the model pay more attention to difficult-to-classify samples.

9 $$Dice=\frac{{2\left| {A \cap B} \right|}}{{\left| A \right|+\left| B \right|}}$$

10 $$Dice\_Loss=1 - Dice$$

Where *A* is the segmentation result predicted by the model, and *B* is the real label of the image. The *Dice* coefficient is in the range of [0,1], and the closer to 1, the more accurate the prediction is.

### **Experimental results and analysis of Blade-Deeplabv3 + blade region segmentatio**n

**Fig. 4:**
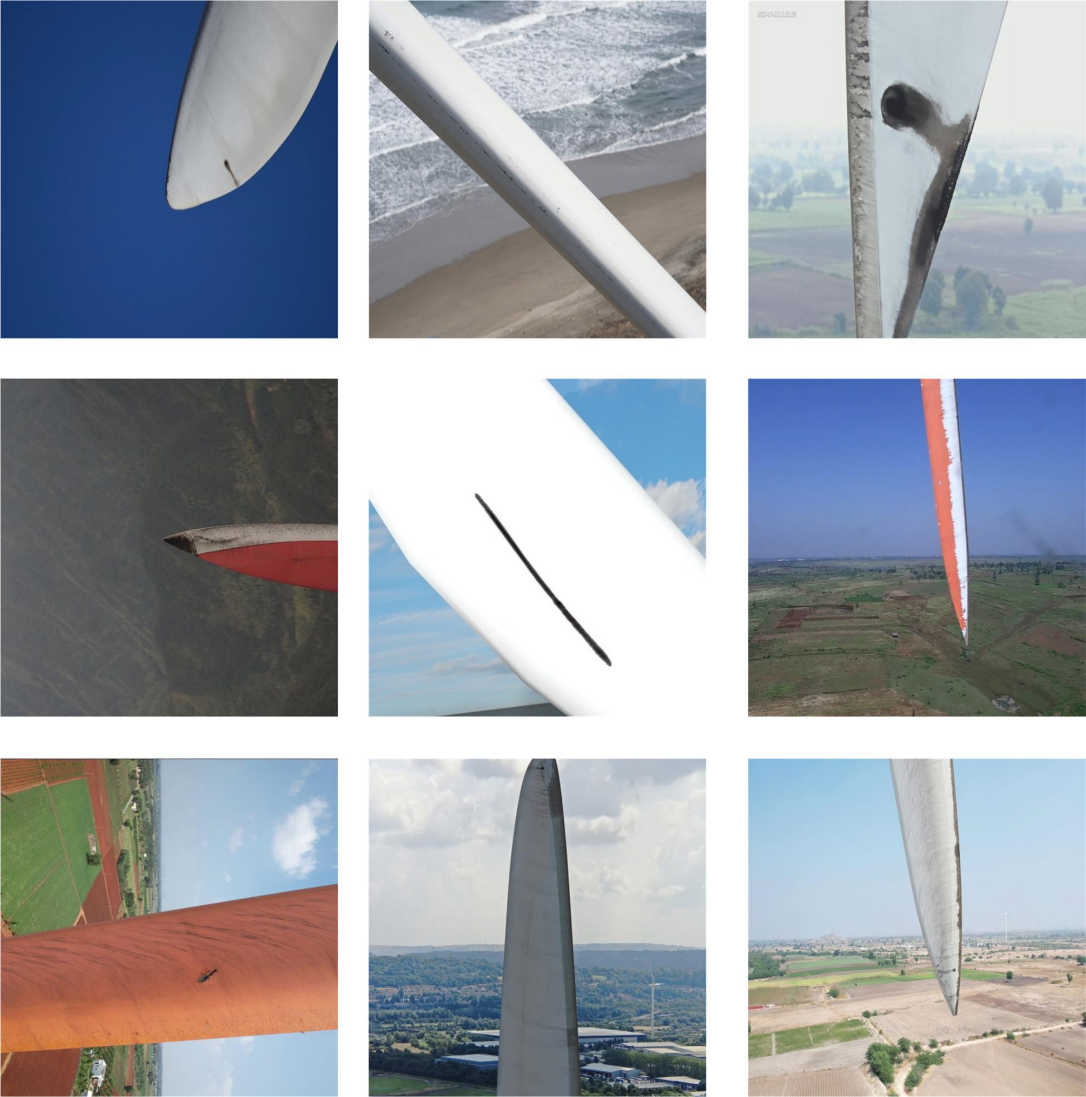
Part of the blade area segmentation picture example.

The blade region is segmented. There are 4325 images in the original image data set of high-resolution blades taken by drones. The dataset used in this study was constructed from images selected from two publicly available sources: AIrotor Less Number of Classes^32^ and Wind Turbine Blade Surface Defect Dataset^33^, with manual annotations to generate ground truth masks. The training phase is divided into a training set and a verification set according to 9:1. The original data set contains a variety of backgrounds. The shooting angle and weather changes make the image of the data set show significant differences, and the shape, size, light and darkness, and color of the blades are different, improving the data set’s diversity. To reduce the impact of noise on the image segmentation model, data augmentation techniques including brightness adjustment, horizontal flipping, and random scaling were applied to simulate variations in camera viewpoint, rotation, and object scale. These augmentations enhance the model’s robustness to noise and variations in blade appearance, improving its generalization capability. Part of the blade area segmentation image is shown in Fig. 4.

To evaluate the performance of the deep learning model, this study uses precision, Accuracy, and MIoU as metrics. The prediction results can be classified into four cases: correctly identifying positive samples as positive samples, that is, TP (True Positive); the positive samples are mistakenly identified as negative samples, namely FN (False Negative); negative samples are mistakenly identified as positive samples, namely FP (False Positive); and correctly identify negative samples as negative samples, namely TN (True Negative).

11 $$Accuracy=\frac{{TP+TN}}{{TP+FP+TN+FN}}$$

12 $$\Pr ecision=\frac{{TP}}{{TP+FP}}$$

13 $$MIoU=\frac{1}{n}\sum\limits_{1}^{i} {\frac{{TP}}{{TP+FP+FN}}}$$

The wind turbine blade region segmentation experiment compares the performance of Deeplabv3+, U-Net, and PSPNet under different backbone networks. Table 4 shows the segmentation performance of each model. Deeplabv3+, which uses Xception as the backbone network, has the highest accuracy in the background and blade region segmentation task. This model is selected as the first stage model of two-stage blade surface defect segmentation.

**Table 4:** Comparison of regional segmentation performance of blades of each model.

Model	IoU		MIoU	Precision	mPA
	Background	Blade			
DeepLabv3+(Xception)	98.65%	99.29%	98.97%	99.49%	99.48%
DeepLabv3+(MobilenetV2)	98.43%	99.18%	98.81%	99.41%	99.39%
U-Net(resnet50)	93.59%	96.64%	95.12%	97.74%	97.26%
U-Net(VGG16)	96.58%	98.20%	97.39%	98.79%	98.57%
PSPNet(resnet50)	97.89%	98.89%	98.39%	99.27%	99.11%
PSPNet(MobilenetV2)	97.85%	98.87%	98.36%	99.20%	99.14%

**Fig. 5:**
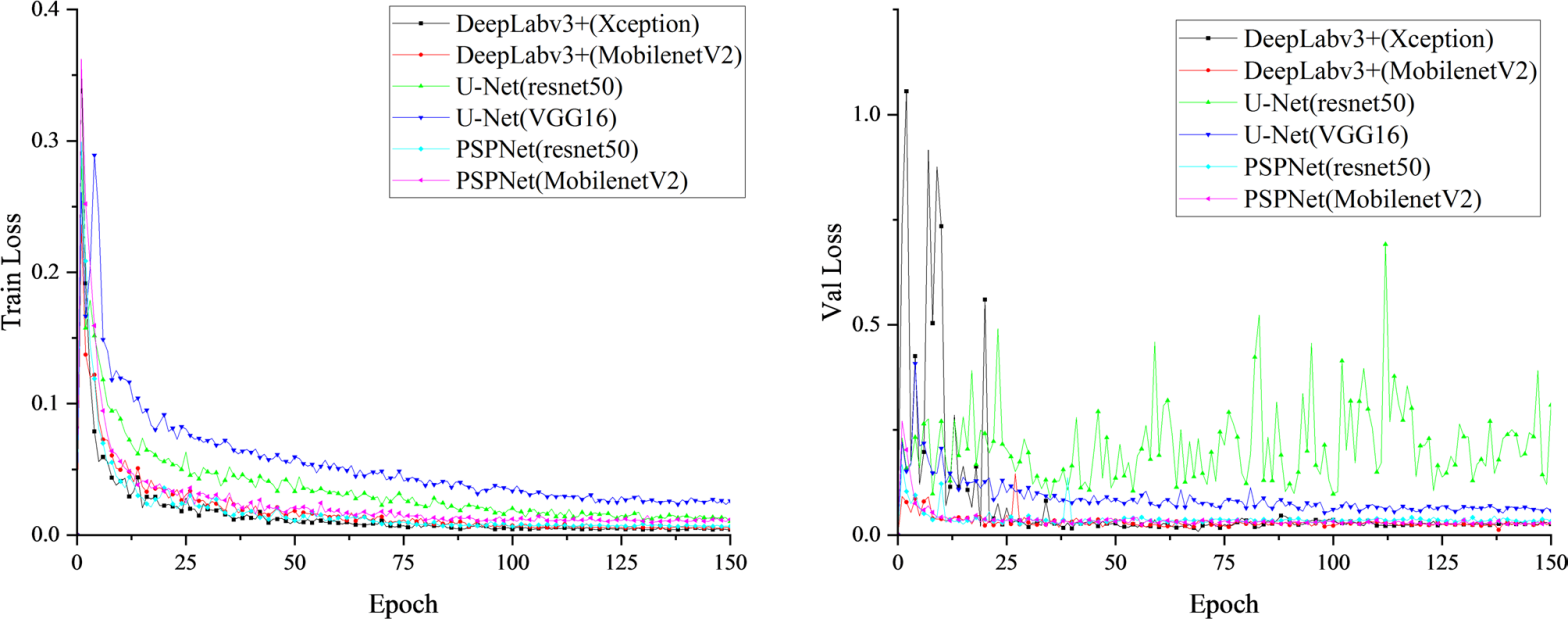
Blade region segmentation training loss function curve of each model.

**Fig. 6:**
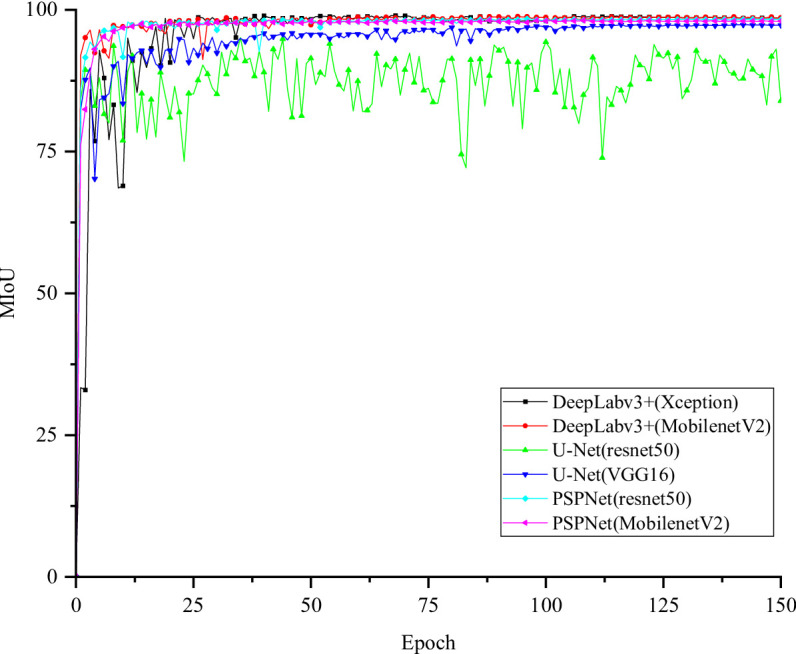
MIoU curve training for each model of blade region segmentation.

Figure 5 shows the training and validation loss curves of different segmentation models over each epoch. All models exhibit steadily decreasing losses and eventual convergence, indicating stable optimization. Deeplabv3+ (Xception) converges faster and achieves the lowest final loss, reflecting superior feature extraction from complex blade regions. Its validation loss remains stable after convergence, demonstrating strong generalization and resistance to overfitting. The corresponding mIoU curves are presented in Fig. 6. After 40 epochs, Deeplabv3+ (Xception) attains the highest mIoU with minimal fluctuations, whereas the U-Net series models display larger oscillations and less stable optimization. Overall, these results confirm that Deeplabv3+ (Xception) outperforms other models in first-stage wind turbine blade region segmentation, achieving an mIoU of 98.97%.

**Fig. 7:**
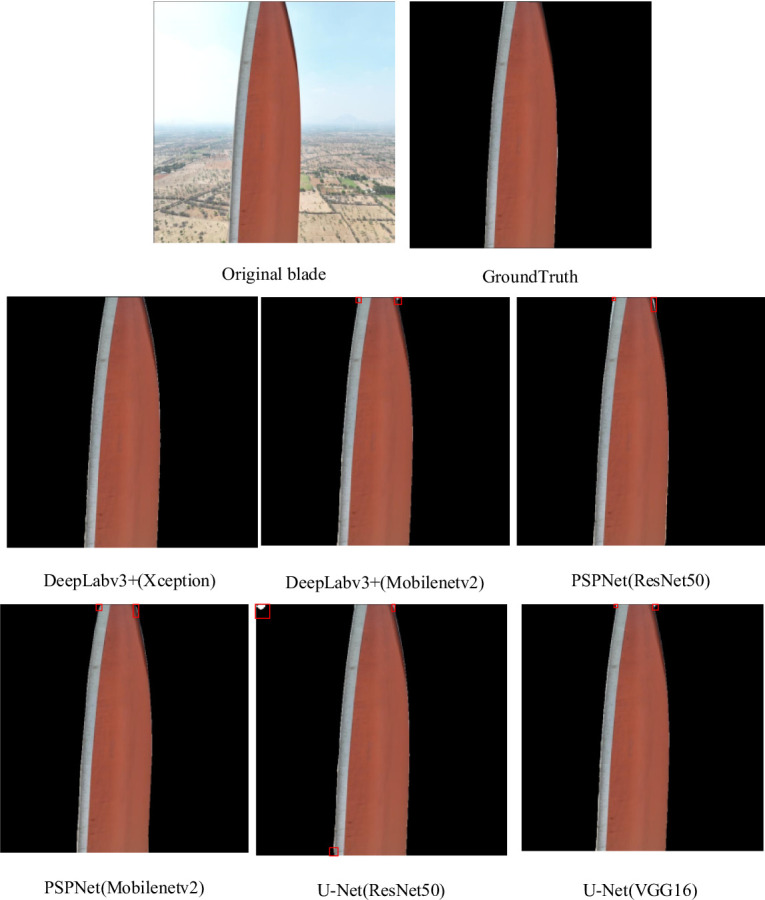
Blade segmentation results of each model.

The segmentation results of each model are shown in Fig. 7. Each model performs well in the first stage of blade and background segmentation tasks. Deeplabv3 + using Xception as the backbone network has the best effect among them. The other models have poor segmentation results due to their different network depth and feature extraction capabilities. Among them, U-Net (ResNet50) misunderstands the background as the blade area, such as the area marked by the red box in the figure.

### Defect-Deeplabv3 + blade defect region segmentation experimental results and analysis

The dataset used to segment the defect area consists of images processed in the first stage by Blade-Deeplabv3+, where the background is removed and only blade regions with visible defects are retained. The defect samples include 1303 crack images, 1093 hole images, and 1054 spalling images, all manually annotated to provide pixel-level ground truth masks. The improved Deeplabv3+ (Xception) with the DenseASPP module is selected as the main model for the blade defect segmentation experiment. This part of the study compares six different models, including variants with different backbone networks, and the IoU values for each defect type obtained by each model are presented in Table 5.

**Table 5:** Comparison of IoU segmentation of blade defects in each model.

Model	IoU				MIoU
	Background	Crack	Hole	Spalling	
DeepLabv3+(Xception)	99.77%	86.09%	92.18%	97.85%	93.98%
DeepLabv3+(MobilenetV2)	99.75%	85.89%	92.01%	97.55%	93.80%
U-Net(resnet50)	99.67%	82.20%	84.23%	96.18%	90.57%
U-Net(VGG16)	99.70%	84.92%	92.17%	96.69%	93.37%
PSPNet(resnet50)	99.65%	75.11%	92.02%	97.44%	91.05%
PSPNet(MobilenetV2)	99.59%	71.30%	89.21%	97.08%	89.29%
DeepLabv3+(DenseAspp)	99.79%	86.27%	92.92%	98.04%	94.25%
Swin-UNet	99.46%	77.30%	63.95%	93.62%	83.59%
Trans-UNet	99.73%	85.46%	92.77%	97.14%	93.78%

**Fig. 8:**
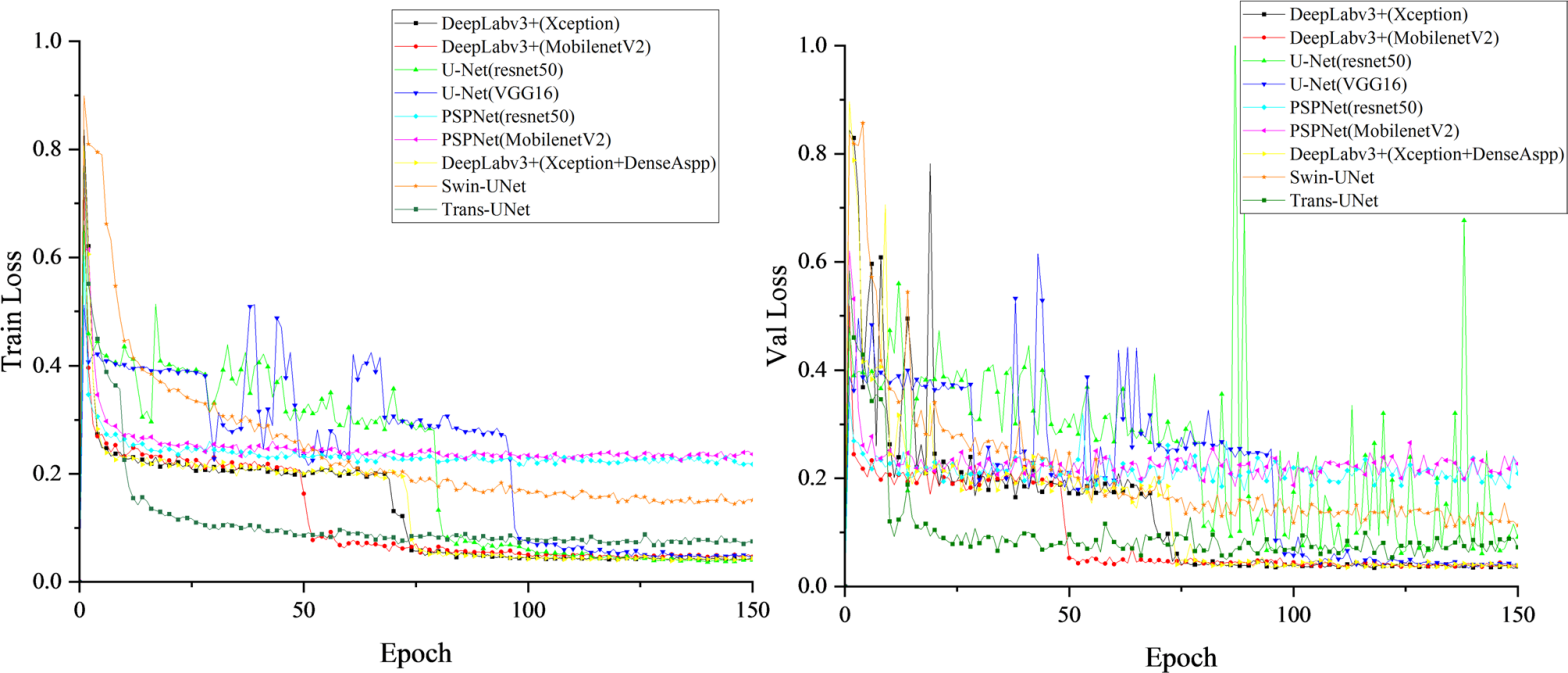
Defect segmentation training loss function curve of each model.

**Fig. 9:**
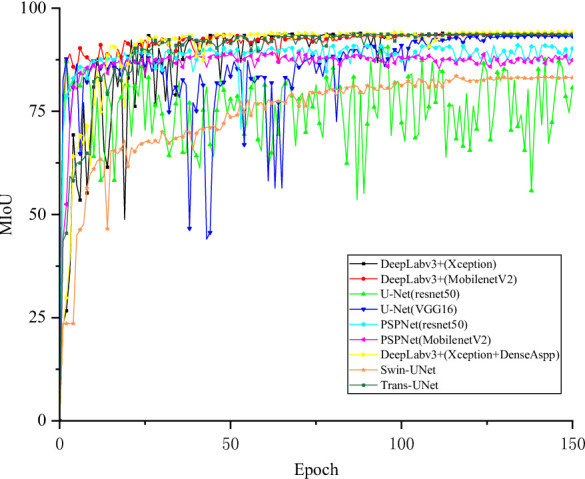
Defect segmentation Each model trains the MIoU curve.

**Fig. 10:**
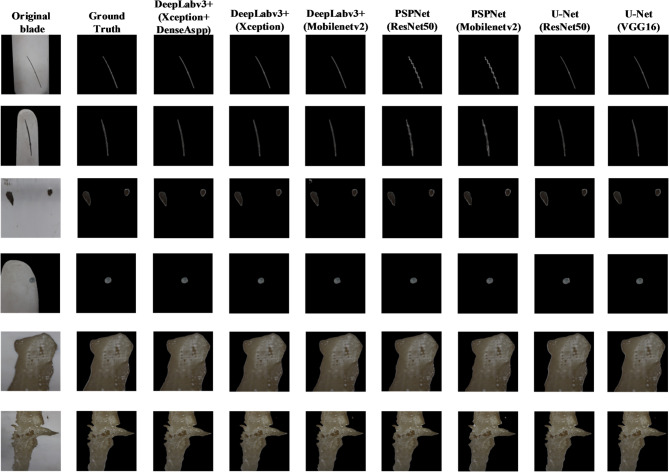
defect segmentation results of each model.

In the second stage of the blade surface defect segmentation model, the loss function change curve corresponding to each model is shown in Fig. 8, and the MIoU change curve of different models is shown in Fig. 9. After the 75th Epoch, the Deeplabv3+ (Xception) loss function improved by DenseAspp gradually decreased and tended to be stable. The MIoU of the Deeplabv3+ (Xception) model improved by DenseAspp reached 94.25%, which was 0.27% higher than that of the original Deeplabv3+ (Xception). The corresponding IoU of various defects was also improved. Among them, the minor target defect of the hole was the most significant, at 0.74% higher than that of the original model. The defect segmentation model training MIoU curve is shown in Fig. 10.

Figure 10 shows the defect segmentation results of each model. Each model has a good segmentation effect for large-area damage defect types. However, other models easily misjudge holes as damaged areas except for Deeplabv3+ (Xception), which DenseAspp improved. PSPNet has the worst segmentation effect on crack defect types, including too many blade areas.

### Grading evaluation of blade surface defect severity

**Fig. 11:**
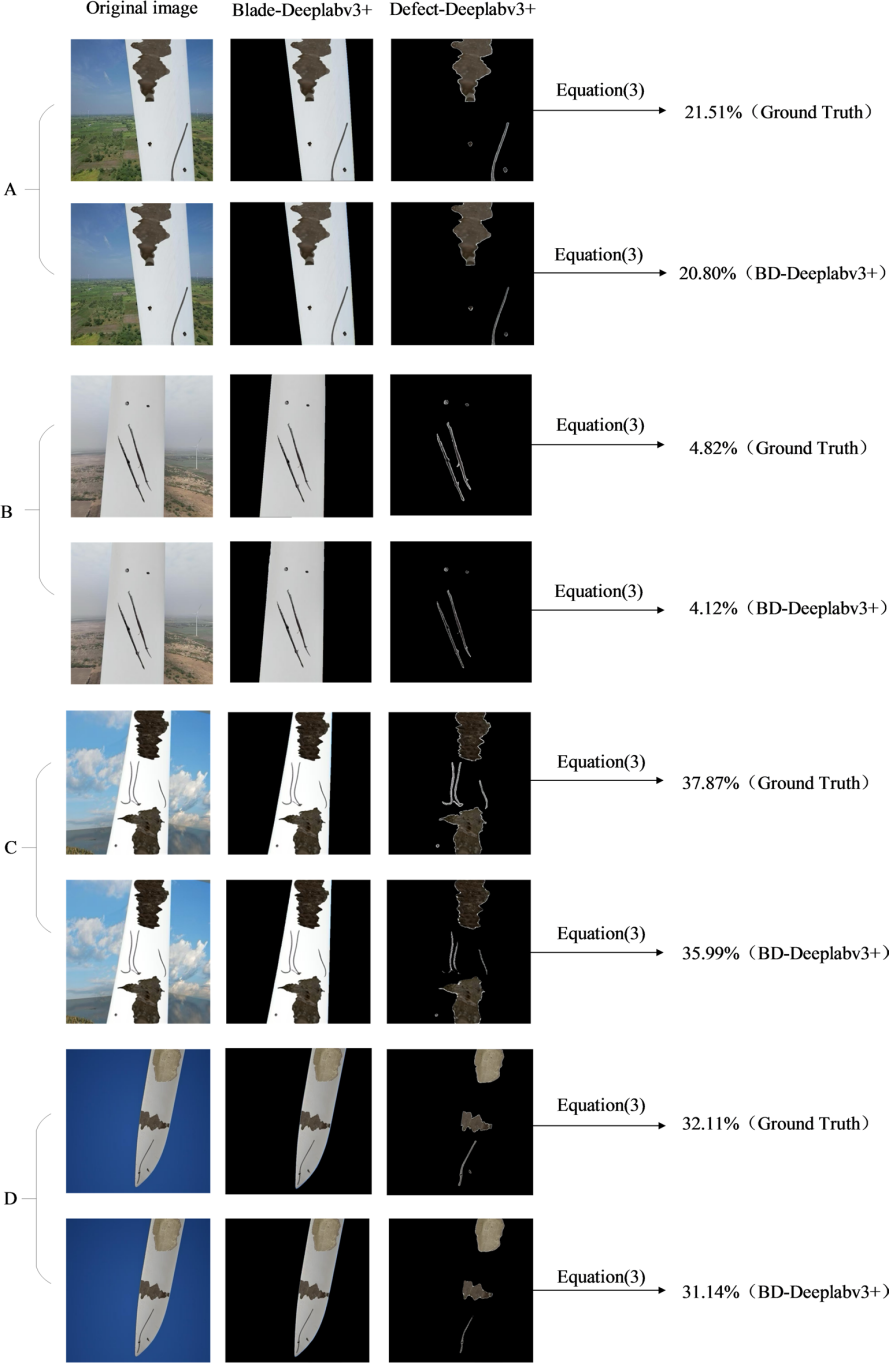
Grading assessment of severity of blade defect area.

Grading assessment of the severity of blade defects by calculating the ratio of the area of the defect area segmented by the BD-Deeplabv3 + model to the area of the blade area, the defect part in the picture accounts for less than 15% of the blade area and is divided into risk level 3 categories. The defects that account for 15% to 30% are divided into risk level 2 categories, and more than 30% are divided into risk level 1 categories. The classification evaluation standard refers to the classification method of bridge structure safety evaluation after an earthquake disaster in the civil engineering field^34^. Table 6 lists the background of the original image of each blade defect, the proportion of the area of the blade, and various defects. The proportion of the area of the blade defect and the BD-Deeplabv3 + segmentation result is shown in Fig. 11. Comparing the segmentation results in Fig. 11-C with the actual situation, BD-Deeplabv3 + has insufficient segmentation of cracks with curved geometry, resulting in a smaller proportion of defect areas in the segmentation results than the actual defect areas. In Fig. 11-D, BD-Deeplabv3 + ignores the segmentation of the hole area, resulting in a smaller segmentation result.

**Table 6:** Evaluation results of blade surface defect severity grading.

The original picture	Proporation						Severity
	Background	Blade	Crack	Hole	Spalling	Overall	
A(Ground Truth)	59.98%	40.02%	0.72%	0.15%	7.74%	21.51%	2
A(BD-Deeplabv3+)	59.71%	40.29%	0.61%	0.14%	7.64%	20.80%	2
B(Ground Truth)	58.92%	41.08%	1.86%	0.12%	0%	4.82%	3
B(BD-Deeplabv3+)	58.72%	41.28%	1.54%	0.16%	0%	4.12%	3
C(Ground Truth)	65.30%	34.70%	1.12%	0.04%	11.98%	37.87%	1
C(BD-Deeplabv3+)	65.44%	34.56%	0.61%	0.03%	11.80%	35.99%	1
D(Ground Truth)	81.16%	18.84%	0.38%	0.03%	5.64%	32.11%	1
D(BD-Deeplabv3+)	81.34%	18.66%	0.24%	0%	5.57%	31.14%	1

## Conclusion

A two-stage wind turbine blade defect segmentation method based on Deeplabv3 + is proposed to address challenges posed by complex background interference and small, irregular defect features. The main conclusions are as follows:

(1) In the first stage, background noise is effectively suppressed, enabling accurate segmentation of the blade body, with a MIoU of 98.97%.

(2) In the second stage, the traditional ASPP is replaced by the DenseASPP module with densely connected dilated convolutions, improving segmentation of cracks, holes, and spalling defects. The defect area MIoU reaches 94.25%, and the MIoU for hole defects increases to 92.98%, demonstrating the effectiveness of dense multi-scale feature fusion for characterizing small defects.

(3) Based on the two-stage segmentation results, a preliminary defect severity assessment is performed using the defect-to-blade area ratio. Comparison with finely labeled ground truth confirms that the model accurately segments defects and enables reliable classification and evaluation.

Future work will focus on improving the model’s robustness to noise, which includes exploring advanced data augmentation techniques, noise-robust loss functions, and more refined post-processing methods to ensure reliable performance under varying noise conditions in real-world applications. Although this study primarily focuses on improving image segmentation for wind turbine blade surface defect detection, analyzing the model’s computational complexity remains an important aspect. Due to the scope of the current work, this aspect was not addressed; future research will investigate it to more comprehensively evaluate the model’s efficiency and resource requirements.

## Data Availability

The data that support the findings of this study are available from the corresponding author upon reasonable request.

## Abbreviations

ASPP: Atrous spatial pyramid pooling
DenseASPP: Densely connected atrous spatial pyramid pooling
DINO: DETR with Improved denoising anchor boxes
ECA: Efficient channel attention
PSA: Polarized self-attention
BN: Batch normalization
ReLU: Rectified linear unit
Conv: Convolutional layer
YOLO: You only look once
ResNet: Residual network
VGG: Visual geometry group network
MobileNet: Mobile neural network
Xception: Extreme inception
PSPNet: Pyramid scene parsing network
MIoU: Mean Intersection over Union
MPA: Mean pixel accuracy
